# Single-stage posterior-only debridement, decompression and interbody fusion for the treatment of thoracolumbar spinal tuberculosis complicated with psoas abscesses

**DOI:** 10.1186/s12893-021-01092-8

**Published:** 2021-02-12

**Authors:** Wence Wu, Zhechen Li, Renqin Lin, Shenglin Wang, Jianhua Lin

**Affiliations:** Department of Spinal Surgery, The First Affiliated Hospital of Fujian Medical University, Chazhong Road 20th, Fuzhou, 350005 Fujian China

**Keywords:** Single-stage, Posterior-only, Debridement, Thoracolumbar spinal tuberculosis, Psoas abscesses

## Abstract

**Background:**

To explore the clinical safety and efficacy of single-stage posterior-only debridement, decompression, allograft bone using titanium mesh and interbody fusion combined for the treatment of thoracolumbar spinal tuberculosis complicated with psoas abscesses.

**Methods:**

A total of 38 patients diagnosed with thoracolumbar spinal tuberculosis complicated with psoas abscesses underwent surgery via single-stage posterior-only debridement, decompression, allograft bone using titanium mesh and interbody fusion from January 2010 to September 2016 were enrolled in the study. The clinical efficacy of the approach was assessed based on parameters including operating time, blood loss, Cobb angle, visual analogue scale (VAS) scores, Frankel grade, erythrocyte sedimentation rate (ESR) and C-reactive protein (CRP).

**Results:**

The surgery duration was 224.4 ± 71.1 min with a blood loss of 731.8 ± 585.8 ml. The Cobb angle was corrected from 16.0 ± 15.4° preoperatively to 8.1 ± 7.4° postoperatively (P < 0.001, t = − 4.38), and returned to a level of 11.0 ± 8.5° at the final follow-up (P = 0.002, t = 3.38). Back pain was relieved, with the mean preoperative VAS of 3.5 ± 1.1 decreased to 0.7 ± 0.8 postoperatively (P < 0.001, t = 23.21) and then to 0.6 ± 0.5 at the final follow-up (P < 0.001, t = 17.07). Neurological function was improved in various degrees and psoas abscesses disappeared in all patients. The ESR and CRP decreased gradually after surgery and returned to normal at the final follow-up in all patients. All patients achieved bone fusion thoroughly and no recurrence of TB or surgical related complications was found at the final follow-up.

**Conclusion:**

Single-stage posterior-only debridement, decompression, allograft bone using titanium mesh and interbody fusion is a safe and effective approach for the management of thoracolumbar spinal tuberculosis complicated with psoas abscesses.

## Introduction

Spinal tuberculosis (TB), the most common form of extra pulmonary TB, is a severe spinal disease that frequently causes severe kyphosis and permanent paralysis [1]. The pathophysiology includes tuberculosis spreading into the vertebrae, limitation of nutrient flow, and finally destruction of the vertebrae [2]. The thoracolumbar spine is the major target of TB [3], with approximately 75% of the cases accompanied with psoas abscesses [4]. With the advent of anti-TB chemotherapy, most thoracolumbar spinal TB patients can achieve satisfactory results by conservative treatment only. However, psoas abscesses largely negate the efficacy of anti-TB chemotherapy and surgical treatments are demanded for those cases. The aim of surgery is to achieve thorough debridement, adequate nerve decompression and reconstruction of spinal sequence. The management of the posterior approaches for patients with spinal tuberculosis was widespread and brought out favorable outcomes [5, 6]. And there are some literatures on the treatment of thoracolumbar spinal TB with psoas abscesses via percutaneous drainage or combined anterior–posterior approach [7]. However, to our knowledge, there are few reports on one-stage posterior-only approach for the treatment of thoracolumbar spinal TB with psoas abscesses. Therefore, this present study was designed to explore the clinical safety and efficacy of single-stage posterior-only debridement, decompression, allograft bone using titanium mesh and interbody fusion for the treatment of thoracolumbar spinal tuberculosis complicated with psoas abscesses.

## Methods

### Study patients

The retrospective study was approved by the ethics board committee of The First Affiliated Hospital of Fujian Medical University. In addition to this, we confirm that all methods were performed in accordance with the relevant guidelines and regulations. A total of 38 patients diagnosed with thoracolumbar spinal tuberculosis with psoas abscesses were enrolled in this retrospective study. All patients included underwent single-stage posterior-only debridement, decompression, allograft bone using titanium mesh and interbody fusion from January 2010 to September 2016. The diagnosis of thoracolumbar spinal TB was confirmed based on clinical manifestation, such as back pain, slight fever, night-sweats or weight loss; the positive laboratory results of tuberculin test and T-SPOT test, and increased levels of erythrocyte sedimentation rate (ESR) and C-reactive protein (CRP); vertebrae destruction and psoas abscesses observed by plain films, computed tomography (CT) and magnetic resonance imaging (MRI); and sequestrum and granulation tissue observed by pathological examination. The Frankel grading system was used to evaluate the neurological function of patients. The back pain was recorded using visual analogue scale (VAS) scores. Various parameters including Cobb angle, operative time, blood loss, erythrocyte sedimentation rate (ESR), C-reactive protein (CRP) and postoperative complications were used to evaluate the clinical effectiveness of the surgery.

### Preoperative care

Patients enrolled in this retrospective study received preoperative chemotherapy of HREZ with a dosing consisted of isoniazid (H, 300 mg/d), rifampicin (R, 450 mg/d), ethambutol (E, 750 mg/d) and pyrazinamide (Z, 750 mg/d) for at least two weeks. Surgical treatment was performed when the patient got relieved typical symptoms, decreased ESR and CRP levels, and recovered hypoproteinemia.

### Surgical techniques

After general endotracheal anesthesia, patients were placed in the prone position. Through posterior midline incision, the posterior tissues were exposed, centered on the diseased vertebral body and extended to one or two vertebrae upward and downward. Then transpedicular screws were installed at the mild side of vertebral pedicle based on the symptoms and imaging results. Before debridement and decompression, a temporary rod was stabilized in order to protect spinal cord from injury. The severe side of the abscess was performed with debridement of the affected vertebrae, necrotic disc and paravertebral abscess. Then, according to the range of abscess, we performed debridement through one or both pedicles. After eliminating the affected vertebrae, necrotic disc and paravertebral abscess, a flush tube was inserted in psoas abscess to wash the focus with appropriate pressure until no sanies outflow. Then, levofloxacin was used to wash the lesion again. After that, titanium mesh filled with allograft bone was used to rehabilitate spinal sequence, with a transpedicular screw and rod system to achieve the stability of spine. Lastly, streptomycin (1 g) and isoniazid (0.3 g) were placed in the operation site. To achieve improved focus debridement, two drainage tubes were inserted in the abscess cavity before the incision was closed.

### Postoperative care

When the drainage volume was less than 30 ml/24 h, the drainage tubes were removed. Postoperative patients received treatments of mannitol dehydration (375 ml/day) and conventional nerve nutrition (mecobalamin, 1.5 mg/day) to reduce any postoperative edema of the incision and nerves, and to accelerate neurological recovery. Patients were encouraged to exercise their limbs in bed and were instructed to wear orthosis until the achievement of bone fusion. Anti-TB chemotherapy was continued post-operatively for patients with the same as preoperative regimen for six months, followed by another 9–12 months regimen of isoniazid, rifampicin and ethambutol. All patients underwent periodical radiological and laboratory examination at 1 week, and 3, 6, and 12 months after surgery, and annually thereafter.

### Statistical analysis

All measurement data were expressed as mean ± SD. The SPSS 21.0 software (IBM Corp., Armonk, NY, USA) was used for statistical analyzation. Pre- and postoperative comparisons were performed by the Paired-sample t-test, with a P value < 0.05 regarded as statistically significant.

## Results

### Baseline

The characteristics of 38 patients were shown in Table 1. Twenty-three (60.5%) males and 15 (39.5%) females with average age of 46.9 ± 18.1 years (range, 21–77 years) were included in the present study. There were 26 patients (68.4%) < 60 years old and 12 patients (31.6%) ≥ 60 years old. Among them, 9 patients (23.7%) and 12 patients (31.6%) suffered from fever and night-sweats respectively. Four patients (10.5%) got weight loss (5–10 kg). Thirty-four patients (89.5%) complained of back pain. Thirty patients (78.9%) were found to be involved in two vertebrae, with the other six (15.8%) and two (5.3%) involved in three and four vertebrae respectively. Involved levels were observed at T11-T12 in 14 cases, T11-L1 in six cases, T11-L2 in two cases, T12-L1 in seven cases and L1-L2 in nine cases. Psoas abscess was observed in all patients, including 12 patients on the left side, 8 on the right side, and 18 on both sides.

**Table 1:** Clinical data of all patients

Basic demographic data	n (%)
Sex	
Male	23 (60.5)
Female	15 (39.5)
Age (years), mean (range)	46.9 (21–77)
< 60	26 (68.4)
≥ 60	12 (31.6)
Low-grade fever	
None	29 (76.3)
Yes	9 (23.7)
Night-sweats	
None	26 (68.4)
Yes	12 (31.6)
Weight loss (kg)	
0	21 (55.3)
> 0 and ≤ 5	13 (34.2)
> 5 and ≤ 10	4 (10.5)
Back pain	
None	4 (10.5)
Yes	34 (89.5)
Number of affected vertebrae	
2 segments	30 (78.9)
3 segments	6 (15.8)
4 segments	2 (5.3)
Psoas abscess	
Left	12 (31.6)
Right	8 (21.0)
Both	18 (47.4)

### Clinical efficacy

**Fig. 1:**
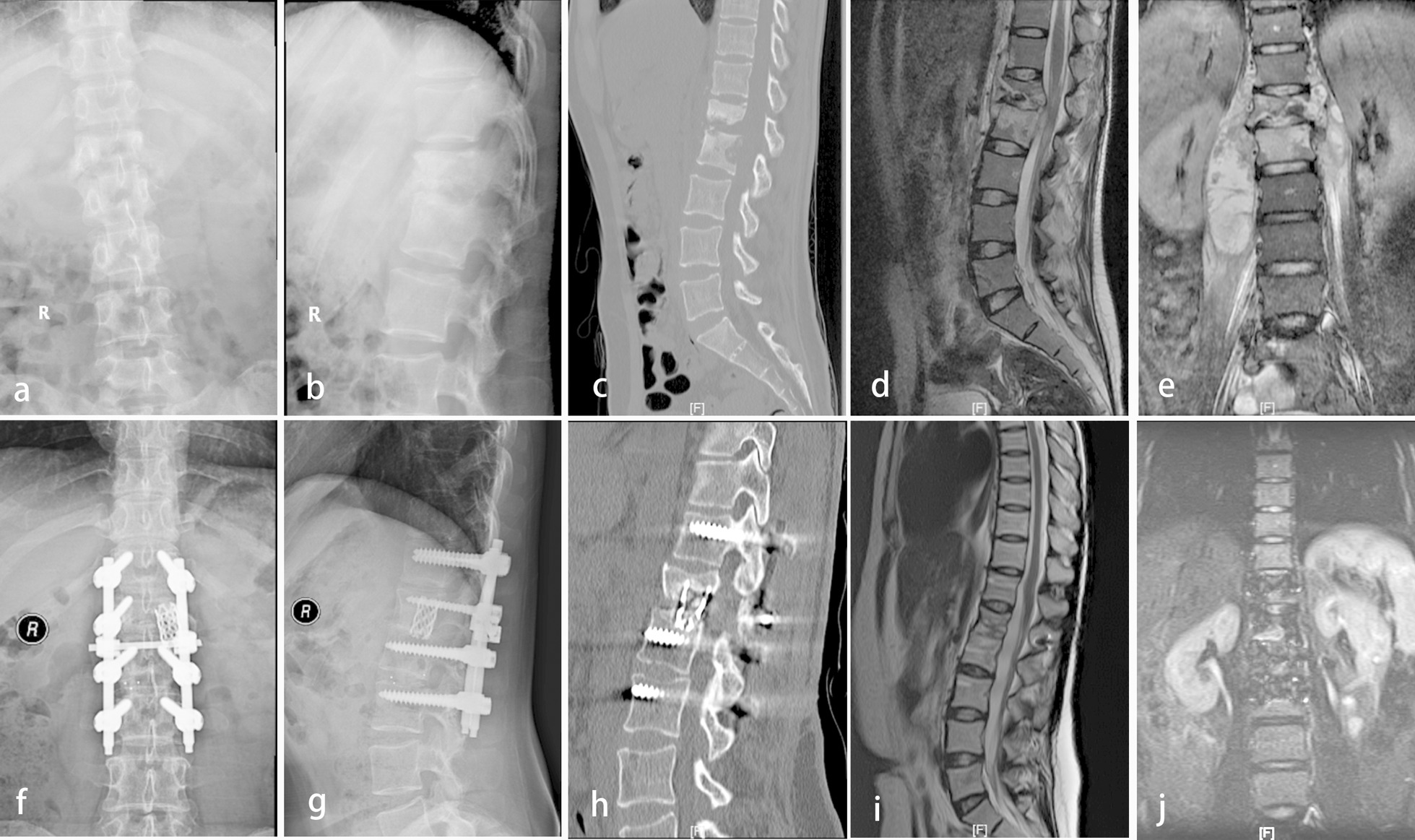
A 27-year-old female patient with thoracolumbar spinal tuberculosis accompany with bilateral psoas abscesses (L1-L2). Preoperative radiography (**a** and **b**), CT (**c**) and MRI (**d** and **e**) showed destruction of anterior column and psoas abscesses. Post-operative radiography (**f** and **g**) and CT (**h**) showed bone fusion achieved with no screw loosening or rod fracture appeared at the final follow-up. Post-operative MRI (**i** and **j**) showed psoas abscesses disappeared at the final follow-up

**Fig. 2:**
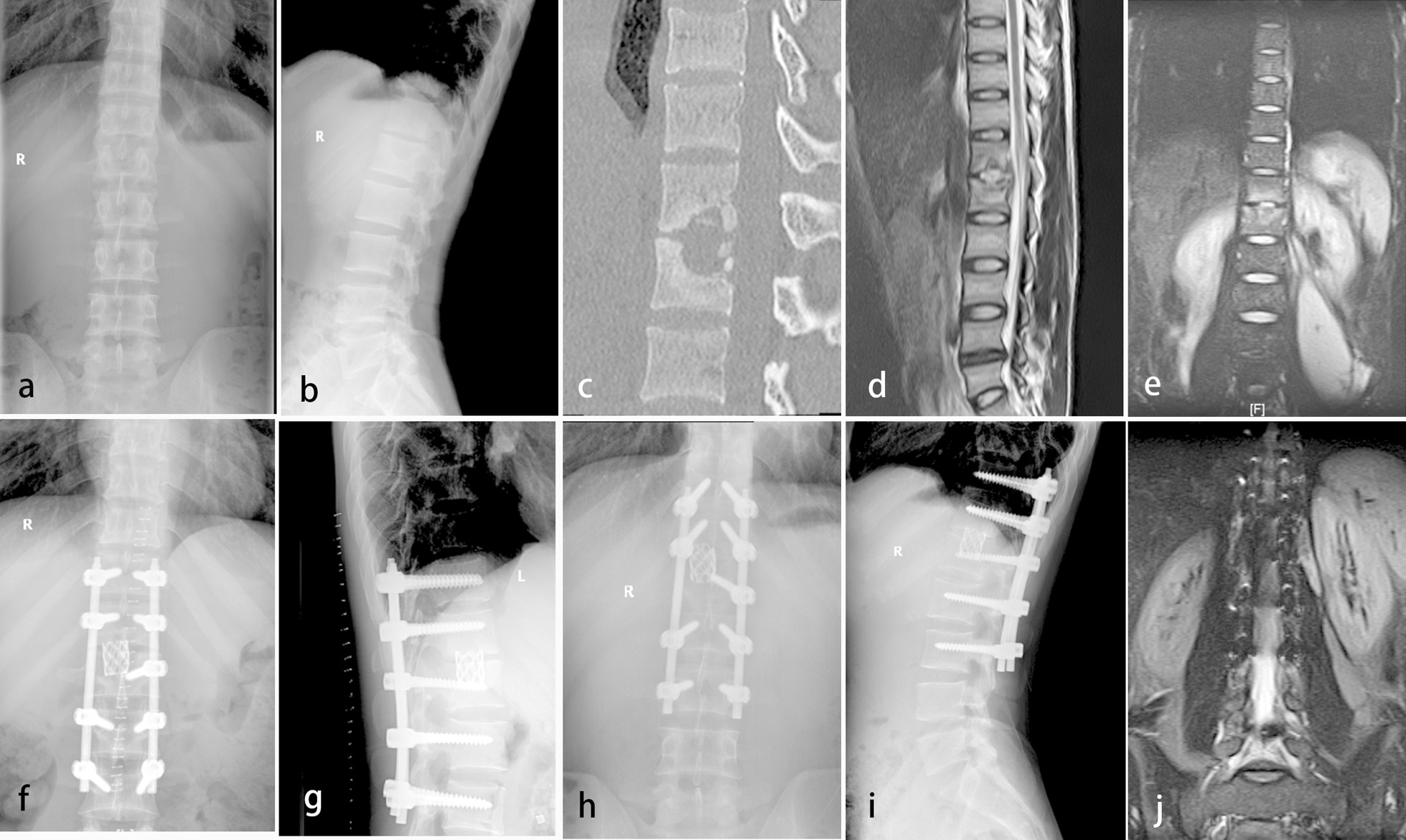
A 28-year-old male patient with thoracolumbar spinal tuberculosis accompany with unilateral psoas abscess (L1-L2). Preoperative radiography (**a** and **b**), CT **(c**) and MRI (**d** and **e**) showed destruction of anterior column and psoas abscesses. Radiography (**f** and **g**) showed no screw loosening or rod fracture appeared three months after surgery. Radiography (**h** and **i**) showed bone fusion at the final follow-up. MRI (**j**) showed psoas abscesses disappeared at the final follow-up

The surgery duration was 224.4 ± 71.1 min, and the blood loss was 731.8 ± 585.8 ml. The mean follow-up duration was 63.2 ± 16.6 months (range, 33–98 months). The preoperative Cobb angle of 16.0 ± 15.4° was corrected to 8.1 ± 7.4° postoperatively, and returned to the level of 11.0 ± 8.5° at the final follow-up. There was significant difference in kyphosis between pre- and post-operative state (P < 0.001, t = − 4.38). While there was 2.9° kyphosis angle loss in average at the final follow-up, the surgery demonstrated significant improvement ability compared with the pre-operative status (P = 0.002, t = 3.38). There was significant back pain relief after the surgery, with the average VAS decreased from 3.5 ± 1.1 preoperatively to 0.7 ± 0.8 postoperatively (P < 0.001, t = 23.21) and then to 0.6 ± 0.5 at the final follow-up (P < 0.001, t = 17.07). The ESR and CRP went down significantly after the surgery (ESR, P < 0.005, t = 3.00; CRP, P < 0.001, t = 5.19) and returned to normal at the final follow up (Table 2). The neurological status got significant improvement in various degrees. Post-operatively, a total of 65.5% patients improved one or two grades, with 66.7% grade B patients increased to grade C (2/3), 54.5% and 9.1% grade C patients increased to grade D (6/11) and E (1/11) respectively, and 58.3% grade D patients increased to grade E (14/24). At the final follow-up, there were up to 92.1% patients improved one or two grades, with all the grade B patients increased to grade D (3/3), all the grade C patients increased to grade D (4/11) or grade E (7/11), and 87.5% grade D patients increased to grade E (21/24) (Table 3). No surgical related complications such as cerebral fluid leakage, nonunion of bone, internal fixation failure, or surgical site infection occurred. No recurrence of TB or sinus tract formation was found in all patients at the final follow-up. All patients achieved bone fusion thoroughly at the final follow-up, with a fusion time ranging from 6 to 9 months (mean 7.4 ± 1.5 months) (Figs. 1 and 2).

**Table 2:** Laboratory findings of the patients

	Pre-op	Post-op	FFU	P1 (t1)*	P2 (t2)*
Cobb angle (°)	16.0 ± 15.4	8.1 ± 7.4	11.0 ± 8.5	< 0.001 (− 4.38)	0.002 (3.38)
VAS	3.5 ± 1.1	0.7 ± 0.8	0.6 ± 0.5	< 0.001 (23.21)	< 0.001 (17.07)
ESR (mm/h)	51.4 ± 25.2	41.9 ± 16.6	18.6 ± 7.7	0.005 (3.00)	< 0.001 (8.85)
CRP (mg/L)	41.9 ± 15.1	30.1 ± 10.8	16.6 ± 7.2	< 0.001 (5.19)	< 0.001 (11.09)

*Pre-op* pre-operative, *Post-op* post-operative, *FFU* final follow-up, *ESR* erythrocyte sedimentation rate, *CRP* C-reactive protein, *P1* post-op versus pre-op, *P2* FFU versus pre-op

*Paired-sample t-test

**Table 3:** Neurological function before and after the surgery

Pre-op Frankel grade	Case number	Frankel grade at post-op					Frankel grade at final follow-up				
		A	B	C	D	E	A	B	C	D	E
A	0	0	0	0	0	0	0	0	0	0	0
B	3	0	1	2	0	0	0	0	0	3	0
C	11	0	0	4	6	1	0	0	0	4	7
D	24	0	0	0	10	14	0	0	0	3	21
E	0	0	0	0	0	0	0	0	0	0	0

*Pre-op* pre-operative, *Post-op* post-operative

## Discussion

The thoracolumbar spine, which represents the transition area from thoracic spine to lumbar spine, is most commonly affected by tubercle bacillus [8]. Patients with thoracolumbar spinal TB often manifest severe vertebrae destruction, kyphosis and compression of spinal cord [8]. Once the disease occurs, the risk of paraplegia is greater than other spinal TB [9, 10]. Due to the peculiar anatomy of the psoas muscle, paraspinal abscess always penetrates the periosteum, and then psoas abscess may be formed in patients with thoracolumbar spinal tuberculosis [2]. Psoas abscesses largely negate the efficacy of anti-TB chemotherapy and surgery is often the preferred treatment modality [11]. Surgical indications for thoracolumbar spinal TB were severe kyphosis or neurological deficit, spinal instability, failed response to chemotherapy, and large psoas abscesses [12]. In the present study, we report our findings regarding the surgical treatment for patients with thoracolumbar spinal tuberculosis via single-stage posterior-only debridement, decompression, allograft bone using titanium mesh and interbody fusion.

Spinal tuberculosis mainly affects the spinal anterior and middle column, leading to kyphosis. Severe kyphosis not only affects appearance, but also causes persistent back pain and late onset paraplegia [13]. Anterior approaches had been successfully applied in the spinal tuberculosis, which is superior at thorough TB focus remove and cord decompression [14, 15]. However, some studies reported that anterior approaches trends to undergo longer surgery duration, larger surgical trauma, more blood loss, neurovascular injury and postoperative complications than posterior approaches during debridement of the abscesses [16, 17]. Posterior interbody fusion and transpedicle screw fixation successfully provides the vertebral stabilization, immediate pain relief and kyphosis correctio [18]. In the present study, the back pain after the surgery was relieved obviously among all patients. The kyphosis angle correction was significant, and the correction satisfactorily suffered from only little kyphosis angle loss. Lee et al. [19] and Güzey et al. [20] reported that spinal tuberculosis patients who suffered from neurological deficit acquire satisfactory functional recovery through posterior debridement and decompression of the spinal cord. In the present study, neurological function was improved in various degrees at the final follow-up.

Guzey et al. [20] and Rath et al. [21] reported that thoracolumbar spinal tuberculosis patients with an abscess could not achieve satisfactory infection debridement and spinal stabilization by posterior-only approach, while anterior approach could gain remarkable effect on the spinal tuberculosis. However, the results of our study declared that thoracolumbar spinal tuberculosis with psoas abscesses could be successfully cured via the posterior-only debridement, decompression, and interbody fusion combined with transpedicle screw fixation. Although ESR and CRP were abnormal in some of them postoperation immediately, because of surgical trauma and the psoas abscess still existing for a period of time, both of them returned to normal levels at the final follow-up.

According to our experience, the successful management of thoracolumbar spinal tuberculosis with psoas abscesses via posterior-only approach could be explained by the following reasons. First, the destruction of vertebrae and disc was the primary focus of tuberculosis, with psoas abscesses as the secondary target. The posterior approach creates enough operating space through the resection of both sides of the facet joint, parapophysis and lamina, allowing 360° direct visualization for thorough removing broken disc, necrotic bone and paraspinal abscess during operation. Second, the psoas abscesses could be effectively eliminated by washing with appropriate pressure using normal saline and anti-TB drugs during operation. Third, posterior-only approach could avoid complications related to the thoracic and abdominal cavity.

The major shortcoming of this study is that the present study is a retrospective investigation rather than a prospective study, which might lead to bias in patient selection. Furthermore, we do not compare the outcomes among anterior-only, combined anterior–posterior and posterior-only approaches. Finally, the results are based on a small group of patients with relatively short follow-up time, more cases with longer follow-up are required to confirm the conclusion drew by this study.

## Conclusions

Through standardized anti-TB therapy, single-stage posterior-only debridement, decompression, allograft bone using titanium mesh and interbody fusion is a safe and effective procedure in the treatment for thoracolumbar spinal tuberculosis with psoas abscesses.

## Data Availability

All data used by or generated in this study is available from the corresponding author upon reasonable request.

## Abbreviations

TB: Tuberculosis
CRP: C-reactive protein
CT: Computed tomography
MR: Magnetic resonance
ESR: Erythrocyte sedimentation rate
VAS: Visual analogue scale
